# Statins and clinical outcome of acute ischemic stroke: a systematic review

**DOI:** 10.1186/1755-7682-3-22

**Published:** 2010-09-29

**Authors:** Shaheen E Lakhan, Sanjit Bagchi, Magdalena Hofer

**Affiliations:** 1 Global Neuroscience Initiative Foundation, Los Angeles, CA, USA

## Abstract

**Background:**

Statin therapy is considered an effective measure for the prevention of ischemic stroke. Several recent studies have indicated that treatment with statins, prior to the onset of acute ischemic stroke, may also substantially reduce the severity of stroke and the degree of patient disability. The purpose of the present review is to systematically evaluate the effectiveness of statin pretreatment on functional outcome of acute ischemic stroke and to assess potential adverse events associated with statin use.

**Methods:**

Relevant articles on the role of statins in acute ischemic stroke were identified via MEDLINE/PubMed, EMBASE, CENTRAL, and by manual searches of the references of identified papers. Clinical studies (most were prospective cohort studies) assessing statin therapy for acute ischemic stroke were selected for the review. Only two randomized controlled clinical trials met the criteria to be included in the analysis. Clinical outcome was assessed based on the degree of disability determined with the modified Rankin Scale (mRS) and Barthel index (BI). The National Institutes of Health Stroke Scale (NIHSS) was used to measure stroke severity. Recurrence of stroke in patients who had suffered from a previous stroke was analyzed with and without statin therapy. Incidence and severity of adverse reactions was reviewed. Because there were too many differences in study outcome measures, a quantitative analysis of data was deemed inappropriate. A qualitative summary of the data was consequently completed.

**Results:**

Thirteen reports were systematically reviewed to evaluate the efficacy and safety of statins in the pretreatment of acute ischemic stroke. Pretreatment with statins was found to reduce the recurrence of stroke and to result in more favorable outcomes for patients. The beneficial effects of prior statin therapy in acute ischemic stroke were shown to be especially profound in whites, diabetics, elderly patients with hypertension and other vascular diseases, and in patients with ideal low density lipoprotein (LDL) levels. There were few incidences of adverse reactions with statin pretreatment, most of which were not statistically significant.

**Conclusions:**

Pretreatment with statins was associated with a favorable outcome in acute ischemic stroke, with few incidences of adverse reactions.

## Background

3-hydroxy-3-methyl-glutaryl-Coenzyme A (HMG-CoA) reductase inhibitors (statins) play an important role in the therapeutic management of coronary heart disease (CHD) as lipid lowering agents [1-6]. Several large, randomized trials have shown that lowering LDL cholesterol with statins reduces coronary mortality and morbidity. The Scandinavian Simvastatin Survival Study, a randomized, double-blind, placebo-controlled trial [1] showed that simvastatin reduced the risk of death by 30% over the median follow-up period of 5.4 years in patients with previous myocardial infarction or stable angina pectoris. In the Heart Protection Study [6], simvastatin given daily over the course of 5 years to high-risk individuals was demonstrated to reduce coronary death rate by 18% compared to placebo. Statin therapy was also shown to prevent major adverse cardiovascular events in asymptomatic subjects at high cardiovascular risk [2,5]. While the role of dyslipidemia in coronary heart disease is clearly well established, the effect of lowering plasma cholesterol levels on the incidence of stroke has been less definitive [7]. Observational cohort studies showed no clear relationship between serum cholesterol and stroke. A meta-analysis of individual data from 61 prospective studies that included a total of 900,000 adults without previous disease did not find a positive and independent association of cholesterol with stroke mortality [8]. However, a meta-analysis of 90,000 patients in previous randomized statin trials showed that the reduction in the risk of stroke was related to the extent to which LDL cholesterol levels were lowered [9]. Recent interventional trials with cholesterol-lowering statins taken for several years show significant decreases in stroke incidence, with relative risk reductions ranging from 20% to 30% [10-12]. Even in the absence of concurrent CHD, the vast majority of patients with previous ischemic stroke may benefit significantly from long-term statin therapy [13].

Studies have also indicated that patients receiving statin therapy before the incidence of stroke suffer less severe strokes and have better clinical outcomes. Although various reports [14-21] and literature reviews [7] have suggested the beneficial role of pretreatment with statin therapy, a systematic review of recent clinical studies is required to evaluate both efficacy and safety of this treatment.

## Methods

The current analysis systematically reviews the efficacy and safety of statins in the pretreatment of acute ischemic stroke, based on previously published research papers.

### Searching for studies

In April 2009, PubMed/Medline, EMBASE, and the Cochrane Central Register of Controlled Trials (CENTRAL) were searched comprehensively using different combinations of the following MeSH and free text terms: acute ischemic stroke, statins, HMG-CoA reductase inhibitors, atorvastatin, simvastatin, pravastatin, fluvastatin, lovastatin, rosuvastatin. Additional studies were identified via the manual searching of references of identified papers. Unpublished data were not sought and abstracts, letters, case reports, and review articles were excluded. (See Additional file 1 for a quality of reporting of meta-analyses (QUOROM) statement checklist.)

### Selection of studies

Clinical studies assessing statin therapy for acute ischemic stroke were selected for the review. Animal studies, studies that did not yield any findings specifically related to ischemic stroke, and studies comparing two different statins without a placebo or treatment control were excluded. Clinical response was defined in terms of the modified Rankin Scale (mRS) and the Barthel Index (BI) which measure patient disability, and the National Institutes of Health Stroke Scale (NIHSS) was used to assess severity of stroke.

The BI evaluates independence in activities of daily living and ranges from severe dependence (score > 40) and assisted independence (score < 60) to a score of 100 which reflects no disability. The mRS assesses functional outcome with scores ranging from 0 to 6 (0, no symptoms; 1, no significant disability despite symptoms and able to carry out all usual duties and activities; 2, slight disability, unable to carry out all previous activities but able to look after own affairs without assistance; 3, moderate disability requiring some help but able to walk without assistance; 4, moderate severe disability, unable to walk without assistance, and unable to attend to own bodily needs without assistance; 5, severe disability; bedridden, incontinent, and requiring constant nursing care and attention; and 6, death). The NIHSS measures stroke severity on a scale of 1-42 with a score of 0 for no stroke, 1-4 = minor stroke, 5-15 = moderate stroke, 15-20 = moderate/severe stroke and 21-42 = severe stroke.

An additional outcome measure was the incidence and severity of adverse events.

### Data abstraction

The authors independently extracted the data, and any disagreements were resolved by consensus. The extracted information from each study included: study type, study objective, sample size, controls, type and amount of stain used, study duration, outcome measures, and reported adverse events.

### Analysis

There were too many differences in outcome measures of studies so a quantitative analysis of data was deemed inappropriate. A qualitative summary of the data was consequently completed.

## Results

### Study characteristics

**Figure 1:**
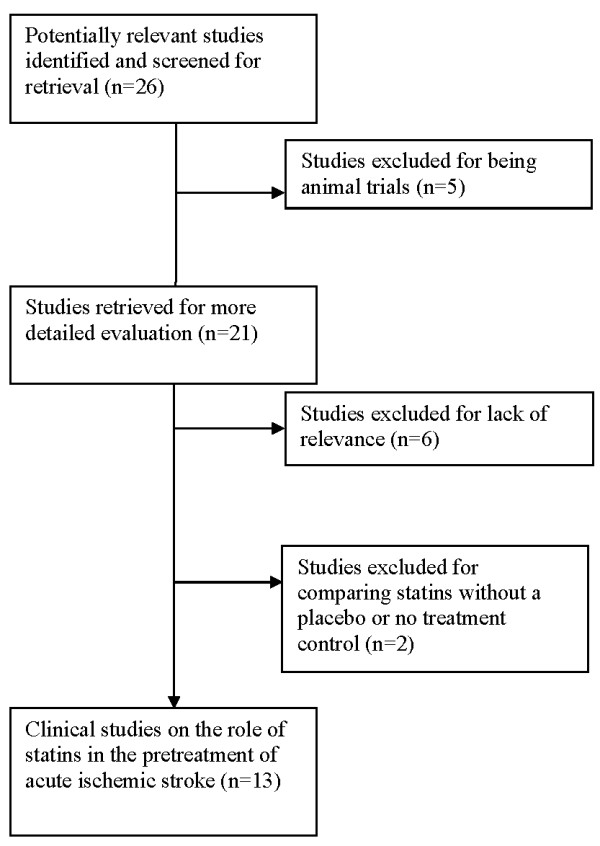
**Flow diagram of included studies**.

Electronic searches found 26 reports that were potentially relevant to the present review. 5 articles were excluded because they discussed animal studies. Six reports were excluded for not yielding any findings specifically related to ischemic stroke. Two studies compared two different statins without a placebo or treatment control and were excluded. (See Figure 1 for a flow diagram of included studies.). Three reports analyzed different patient subtypes or different aspects of the same clinical trial.

A total of 13 studies were reviewed. Eight studies (see Tables 1 and 2) assessed the efficacy of statin therapy [14-21] and 5 articles (Table 3) evaluated the safety of statin therapy [12,22-24]. One report [21] analyzed both efficacy and safety. The length of studies ranged from 1 week to 6 years. Outcome measures included odds ratio (OR), mRS scores, BI and NIHSS scores.

**Table 1:** Clinical outcome in patients taking statins before the incident of stroke

Study	NIHSS	mRS	Other clinical measures (BI, imaging)
Martí-Fàbregas 2004 [14]	Not significantly different	mRS of 0-1 in 80% in statin group vs. 61.3% in non-statin group, P = 0.059	BI of 95-100 in 76.7% in statin group vs. 51.8% in non-statin group, P = 0.015

Yoon 2004 [15]	N/A	51% in statin group vs. 38% in non-statin group had mRS < 2; P = 0.03	

Greisenegger 2004 [16]	N/A	6% in statin group vs. 14% in non statin group had a mRS of 5 or 6	

Moonis 2005 [17]	Significantly more patients had NIHSS ≤ 2 in statin group vs. non-statin, P = 0.008	Significantly more patients had mRS ≤ 2 in statin group vs. non-statin, P = 0.045	

Reeves 2008 [18]	N/A	35.9% in statin group had mRS ≥ 4 vs. 44.3% in non-statin group	

Nicholas 2008	N/A	N/A	Statin pretreatment in patients with diabetes resulted in a smaller than median infarct volume after ischemic stroke (P = 0.01).

Stead 2009 [20]	Not significantly different	Patients on statins were significantly more likely to have a mRS ≤ 2	

Goldstein 2009 [21]	Not significantly different	a trend toward reduced disability with stains based on mRS (P = 0.0647)	

BI, Barthel Index; mRS, modified Rankin Scale; N/A, not available; NIHSS, National Institutes of Health Stroke Scale

**Table 2:** Study design and odds ratio

Study	Study design	# of patients	Follow up period	OR, 95% CI, P value	Measures
Martí-Fàbregas 2004 [14]	cohort	167	3 months	OR = 5.55; 95% CI = 1.42- 17.8; P = 0.012	Favorable outcome (NIHSS, mRS ≤ 2, BI)

Yoon 2004 [15]	observational	436	2 years	OR = 2.9; 95% CI = 1.2-6.7, P = 0.03	Favorable outcome (mRS ≤ 2)

Greisenegger 2004 [16]	cohort	1691	1 week	OR = 0.37; 95% CI = 0.19- 0.74; P = 0.004	Poor stroke outcome (mRS of 5 or 6)

Moonis 2005 [17]	cohort	852	12 weeks	OR = 1.57; 95% CI = 1.04-2.38; P = 0.033	Favorable outcome (mRS ≤ 2)

Reeves 2008 [18]	cohort	1360	6 months	OR 0.74, 95% CI = 0.52-1.02	Poor stroke outcome (mRS ≥ 4 at discharge)

Stead 2009 [20]	cohort	508	22 months	OR = 1.91; 95% CI = 1.05- 3.47	Favorable outcome (NIHSS, mRS ≤ 2)

Goldstein 2009 [21]	Exploratory analysis of SPARCL trial data	576	3 months	N/A	Favorable outcome (NIHSS, mRS)

mRS, modified Rankin Scale; N/A, not available; NIHSS, National Institutes of Health Stroke Scale. OR, Odds Ratio; CI, Confidence Interval

**Table 3:** Adverse events in studies testing statin pretreatment

Study	Study design	# of patients	Follow up period	Findings
PROSPER Study (Shepherd 2002 [22])	Randomized controlled trial	8804	3.2 years	25% increased incidence of cancers (P = 0.020) in patients receiving statin compared to placebo. Meta-analysis of previous pravastatin and other statin trials showed no overall increase in cancer risk.

CTT study (Baigent 2005 [12])	Prospective meta-analysis of 14 randomized trials	90 056	5 years	No evidence of increased the incidence of cancer by statins (OR = 1.00, 95% CI 0.95-1.06; P = 0.9). Excess risk for rhabdomyolysis with statin not significant.

SPARCL Trial (Amarenco 2006 [13],	Double-blind, randomized,	4731	Median 4.9 years	Hemorrhagic stroke was more frequent in statin group, in those with a hemorrhagic stroke as an entry event. Total and LDL cholesterol levels did not affect the risk of hemorrhagic stroke.

Goldstein 2008 [23]	Multicenter trial			

Meier 2009 [24]	Prospective cohort study	311	3 months	More patients with ICH were on statins (30% vs. 15%, P = 0.005). Frequency of ICH is associated with previous statin use (OR = 3.1; 95% CI = 1.53-6.39; P = 0.004).

ICH, intracranial hemorrhage; LDL, Low-Density-Lipoprotein; OR, Odds Ratio; CI, Confidence Interval

### Clinical responses observed

As reported in Martí-Fàbregas (2004), statins may improve long-term outcome when administered before the onset of cerebral ischemia [14]. This prospective study showed that patients who were already being treated with statins at the time of stroke were less affected neurologically and had a better long-term functional outcome than patients who were not receiving statins. Favorable outcomes, defined as a mRS of 0 to 1 and a BI score of 95 to 100 at 3 months were more frequent in the statin group (80% vs. 61.3%, P = 0.059 with the mRS; 76.7% versus 51.8%, *P *= 0.015 with the BI). Mortality was not significantly different in the two groups (3.4% in the statin group and 6.6% in the nonstatin group).

An observational study of 436 patients by Yoon et al (2004) reported that 51% of patients taking statins before hospital admission for stroke had a good outcome at discharge (defined as a Rankin score < 2) compared to 38% of patients not taking statins (P = 0.03). The mortality rate from acute ischemic stroke was 6/84 (7.1%) in patients with statin pretreatment, compared to 30/309 (9.7%) in patients without statin pretreatment [15]. Greisenegger (2004) performed a cross-sectional study of 1691 stroke patients and evaluated clinical severity using the mRS. It was noted that severe acute ischemic stroke (defined as a mRS of 5 or 6) was less frequent in patients receiving statin treatment before the event (6% vs. 14%, OR = 0.37, 95% CI = 0.19-0.74, P = 0.004). Statin pretreatment seems to be associated with reduced stroke-induced disability, most notably in patients with diabetes [16].

Moonis (2005) evaluated the potential effects of statins initiated at least 4 weeks before acute ischemic stroke. Multivariate logistic regression analysis demonstrated that post-stroke statins were associated with a significant probability of a favorable outcome at 12 weeks [NIHSS (P = 0.002; OR = 1.92; CI = 1.27-2.91) and mRS (P = 0.033; OR = 1.57; CI = 1.04-2.38)], whereas pre-stroke statins demonstrated a trend toward significance [17].

Reeves (2008) found that pretreatment with statins was associated with lower odds of poor outcome (OR = 0.74, 95% CI 0.52-1.02) and also reported a significant interaction (P < 0.01) between statin use and race. In whites, statins were associated with statistically significantly lower odds of poor outcome, defined as a modified Rankin score ≥ 4 at discharge (OR = 0.61, 95% CI = 0.42-0.86). In blacks, statins were associated with a non-statistically significant increase in poor outcome (OR = 1.82, 95% CI = 0.98-3.39) [18]. In-hospital mortality was found to be lower among the statin users: 2.3% (n = 7) of the 309 subjects on statins before admission died in-hospital, compared to 6.6% (n = 69) of the 1051 subjects not on statins.

Nicholas (2008) conducted a retrospective cohort analysis of stroke patients to examine infarct size with magnetic resonance diffusion-weighted imaging. They reported a smaller than median infarct volume after ischemic stroke in diabetic patients who were taking statins at onset of ischemic stroke [19].

In a prospective cohort study, Stead (2009) evaluated 508 patients presenting with an acute ischemic stroke and measured their lipid profiles during a 22-month period. Patients with LDL less than or equal to 100 mg/dL and receiving statins (n = 100) were significantly more likely to have a good functional outcome, defined as modified Rankin scale score of 0-2, when compared with those not on the medication (OR = 1.91; 95% CI = 1.05-3.47) [20].

The Stroke Prevention by Aggressive Reduction in Cholesterol Levels (SPARCL) study [13] was the first randomized controlled trial specifically designed to investigate the effect of the reduction in cholesterol levels with atorvastatin in secondary stroke prevention. This trial included a total of 4,731 patients who had a stroke or transient ischemic attack (TIA) 1-6 months before study entry. The patients were randomized to atorvastatin or placebo, with a mean follow-up period of 4.9 years. The results showed a 16% relative risk reduction of recurrent stroke with atorvastatin [13]. Goldstein (2009) performed a secondary analysis of data from the SPARCL study, focusing on the severity and functional outcome of recurrent stroke [21]. There was a trend toward lower disability with treatment based on the mRS score (P = 0.0647) - 218 patients in the atorvastatin group had an ischemic stroke versus 274 in the placebo group - but no difference in stroke severity based on the NIHSS was found.

**Figure 2:**
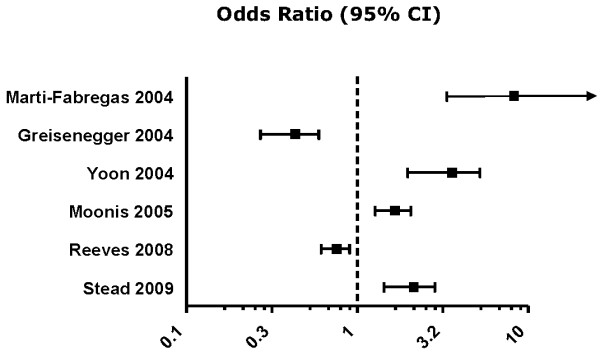
**Odds Ratio for disability based on mRS scores**. Note that Greisenegger 2004 supplied data for the reduced frequency of patients with a higher mRS (enhanced disability) and Reeves 2008 analyzed patients with a mRS ≥ 4, whereas the other studies defined reduced disability as a mRS of ≤ 2.

Overall, the studies reviewed indicated that statin pretreatment results in favorable outcome in stroke patients; however a quantitative analysis of this effect was not feasible because the outcome measures in the studies listed were too variable. An attempt at comparing the quality of the individual studies was made by plotting the OR and 95% CI for reduced disability (mRS) from the studies for which these values were available (Figure 2).

### Adverse reactions observed

Four studies assessed adverse events in patients on statin therapy before the stroke onset (Table 3). In the PROSPER (PROspective Study of Pravastatin in the Elderly at Risk) study, a randomized controlled trial, there was a 25% increased incidence of cancer (P = 0.020) in patients receiving pravastatin for 3 years compared to placebo [22]. However, a meta-analysis of cancer rates in previous pravastatin and other statin trials showed no overall increase in cancer risk. [23]. The CTT study (Cholesterol Treatment Trialists' Collaboration), a prospective meta-analysis of data from 90,056 individuals in 14 randomized trials of statins [12], found no evidence that statins increased the overall incidence of cancer. The 5-year excess risk for rhabdomyolysis with statin was small and not significant. A significant increase of brain hemorrhages in atorvastatin-treated patients was observed in the SPARCL trial in which secondary stroke prevention was analyzed, whereas total and LDL cholesterol levels did not affect the risk of hemorrhagic stroke [13,23]. The risk for brain hemorrhage was also analyzed by Meier (2009) who looked at 311 patients who received intra-arterial thrombolysis [24]. They showed that the overall frequency of intracranial hemorrhage was higher in patients receiving statin therapy before their stroke compared to those without (34.5% vs. 17.6%, P = 0.005). Clinical outcome after 3 months did not differ significantly between the statin and non-statin groups.

## Discussion

The aim of this systemic review was to determine the effect of statin pretreatment on the severity of acute ischemic stroke and on the degree of post-stroke functional disability. Additionally, the safety of statin pretreatment was evaluated. The evidence from cross-sectional, observational and prospective studies as well as randomized placebo-controlled trials was promising, yielding significant findings that suggest that prior statin therapy is associated with favorable outcome and few adverse events.

Statins inhibit HMG-CoA reductase, an enzyme that converts HMG-CoA to mevalonate, a precursor of cholesterol [25]. In acute ischemic stroke, the cholesterol lowering potential of statins comes into action. However, the link between serum cholesterol level and the incidence of stroke remains to be fully established. Several reports [26-28] indicate that in comparison with patients with normal cholesterol levels, patients with high cholesterol levels had a lower risk of death and a lower risk of poor functional outcome. It is becoming clear that the clinical action of many cholesterol-lowering drugs is the result of pleiotropic effects rather than simply a reduction in cholesterol. These pleiotropic effects include attenuation of vascular inflammation, improved endothelial cell function, stabilization of atherosclerotic plaque, decreased vascular smooth muscle cell migration and proliferation, and inhibition of platelet aggregation [28,29]. For example, simvastatin, when administered within 24 hours of the acute ischemic stroke onset, can also inhibit the increase in serum TNF-α, a mediator of inflammation [30] which may have significant clinical impact. There is also evidence, most prominently from the JUPITER trial [31] that statins lower the levels of C-reactive protein (CRP) and may reduce the risk of coronary events independently of the effect of statins on lipid levels. Because increased levels of CRP have been associated with arterial-wall inflammation, statins can prevent ischemia by both inhibiting deposition of lipids and decreasing inflammation.

The stroke protection activity of statins may also be due to the fact that statins improve cerebrovascular reactivity [32].

In acute ischemic stroke, the occurrence and size of cerebral infarction is influenced by the collateral circulation in the brain [33]. Pre-stroke statin use may improve the pretreatment angiographic collateral grade among acute ischemic stroke patients presenting with occlusion of a major cerebral artery. In the cardiovascular system, prior statin treatment enhances the collateralization of coronary arteries and thereby improves the outcome of coronary artery disease. In the cerebrovascular system, statin associated enhancement of collateralization of cerebral arteries leads to a better outcome in acute ischemic stroke. Pretreatment with atorvastatin was associated with better outcome after an ischemic stroke mainly in atherothrombotic and lacunar infarctions, with no significant effect on other stroke subtypes [34].

While pretreatment with statins was associated with better stroke outcomes in whites, there was no evidence of a beneficial effect of statins in black patients [18]. In patients with diabetes, prior statin therapy for acute ischemic stroke was found to be particularly beneficial [16]. The lipid lowering as well as anti-inflammatory effects of statins may have a role in controlling diabetes, which in itself is a risk factor for acute ischemic stroke.

Statin withdrawal, in patients receiving pretreatment with statins for ischemic stroke, was associated at 90 days with increased risk of death or dependency from acute ischemic stroke. This suggests that statin therapy should be continued during the acute phase of ischemic stroke [35].

Few incidences of adverse reactions were reported in clinical studies. The risk of cancer in patients taking statins was evaluated but was found not to be significantly increased [12,22]. Although statins appear to be beneficial for ischemic stroke, the risk for hemorrhagic stroke was increased in patients pre-treated with statins [13,23,24], particularly those with a prior hemorrhagic stroke, male sex, advanced age, and stage 2 hypertension. Therefore, the benefits of statins in preventing ischemic stroke and cardiovascular events have to be weighed carefully against the possibility that they may increase the risk for hemorrhagic stroke.

Prior statin use by patients admitted to hospitals with acute ischemic stroke is increasingly common [15]. This trend can be expected to continue due to continuing reports of the beneficial effects of statins on stroke prevention in elderly individuals with hypertension and vascular disease [36,37]. The American Heart Association/American Stroke Association now recommends the administration of statin therapy with intensive lipid-lowering effects in patients with atherosclerotic ischemic stroke or TIA and without known coronary heart disease to reduce the risk of stroke and cardiovascular events [38,39]

This systematic review concludes that administration of statin treatment before acute ischemic stroke is clinically justified, particularly in whites, diabetics and patients with ideal LDL levels. However, more randomized double-blind placebo-controlled trials are required to definitively evaluate the safety of statin pretreatment, and to compare the efficacy and dosage of individual statin types.

## Abbreviations

BI: Barthel Index; CENTRAL: Cochrane Central Register of Controlled Trials; CHD: coronary heart disease; CI: confidence interval; CR: clinical response; CRP: C-reactive protein; HMG-CoA: 3- hydroxy-3-methylglutaryl coenzyme A; mRS: modified Rankin scale; NIHSS: National Institutes of Health Stroke Scale; OR: odds ratio; QUOROM: quality of reporting of meta-analyses; SE: standard error.

## Competing interests

The authors declare that they have no competing interests.

## Authors' contributions

All authors participated in the preparation of the manuscript, and read and approved the final manuscript.

## Supplementary Material

Additional file 1 **QUOROM statement checklist**. Quality of Reporting of Meta-analyses (QUOROM) statement checklist. Click here for file
